# 

**Published:** 1891-05

**Authors:** 


**Austin District Medical Society.**—The fifteenth quarterly meeting of this society will be held in Austin, on Thursday, June 25, 9:30 a. m.

PAPERS.

"Hysteria," by Dr. Fannie Leake; discussion opened by Drs. J. S. Poyner and B. M. Worsham.

"Typhlitis," by Dr. J. McCaleb; discussion opened by Drs. R. M. Swearingen and W. W. Reeves.

"Amputation of the Cervix Uteri," by Dr. Joseph Cummings; discussion opened by Drs. G. W. Christian and F. R. Martin.

"Empyema," by Dr. Sam. Cunningham; discussion opened by Drs. W. A. Morris and John F. Starley.

"Pessaries," by Dr. T. D. Wooten; discussion opened by Drs. Eugene Clark and J. T. Barnwell.

A proposition will be made soon to exchange a paper occasionally with the different district societies. A member from the Austin Society will visit, say the San Antonio Society, and then the San Antonio Society will return the compliment. In this way the good features of all can be known to each other, and the work of organization promoted.

T. J. BENNETT, Secretary.

